# Silencing MYH9 blocks HBx-induced GSK3β ubiquitination and degradation to inhibit tumor stemness in hepatocellular carcinoma

**DOI:** 10.1038/s41392-020-0111-4

**Published:** 2020-02-14

**Authors:** Xian Lin, Ai-min Li, Yong-Hao Li, Rong-Cheng Luo, Yu-Jiao Zou, Yi-Yi Liu, Chen Liu, Ying-Ying Xie, Shi Zuo, Zhan Liu, Zhen Liu, Wei-Yi Fang

**Affiliations:** 1 Cancer Center, Integrated Hospital of Traditional Chinese Medicine, Southern Medical University, Guangzhou, Guangdong People’s Republic of China 510310; 2 Department of Hepatobiliary Surgery, Affiliated Hospital of Guizhou Medical University, Guiyang, Guizhou People’s Republic of China 550004; 3 Department of Gastroenterology and Clinical Nutrition, The First Affiliated Hospital (People’s Hospital of Hunan Province), Hunan Normal University, Changsha, Hunan People’s Republic of China 410002; 4 Guangzhou Municipal and Guangdong Provincial Key Laboratory of Protein Modification and Degradation, State Key Laboratory of Respiratory Disease, School of Basic Medical Sciences, Guangzhou Medical University, Affiliated Cancer Hospital & Institute of Guangzhou Medical University, Guangzhou, Guangdong People’s Republic of China 510095; 5 Cancer Institute, School of Basic Medical Science, Southern Medical University, Guangzhou, Guangdong People’s Republic of China 510515

**Keywords:** Cell biology, Cancer stem cells, Molecular biology

## Abstract

MYH9 has dual functions in tumors. However, its role in inducing tumor stemness in hepatocellular carcinoma (HCC) is not yet determined. Here, we found that MYH9 is an effective promoter of tumor stemness that facilitates hepatocellular carcinoma pathogenesis. Importantly, targeting MYH9 remarkably improved the survival of hepatocellular carcinoma-bearing mice and promoted sorafenib sensitivity of hepatocellular carcinoma cells in vivo. Mechanistic analysis suggested that MYH9 interacted with GSK3β and reduced its protein expression by ubiquitin-mediated degradation, which therefore dysregulated the β-catenin destruction complex and induced the downstream tumor stemness phenotype, epithelial–mesenchymal transition, and c-Jun signaling in HCC. C-Jun transcriptionally stimulated MYH9 expression and formed an MYH9/GSK3β/β-catenin/c-Jun feedback loop. X protein is a hepatitis B virus (HBV)-encoded key oncogenic protein that promotes HCC pathogenesis. Interestingly, we observed that HBV X protein (HBX) interacted with MYH9 and induced its expression by modulating GSK3β/β-catenin/c-Jun signaling. Targeting MYH9 blocked HBX-induced GSK3β ubiquitination to activate the β-catenin destruction complex and suppressed cancer stemness and EMT. Based on TCGA database analysis, MYH9 was found to be elevated and conferred poor prognosis for hepatocellular carcinoma patients. In clinical samples, high MYH9 expression levels predicted poor prognosis of hepatocellular carcinoma patients. These findings identify the suppression of MYH9 as an alternative approach for the effective eradication of CSC properties to inhibit cancer migration, invasion, growth, and sorafenib resistance in HCC patients. Our study demonstrated that MYH9 is a crucial therapeutic target in HCC.

## Introduction

Hepatocellular carcinoma (HCC) is one of the most commonly diagnosed cancers and the second most common cause of cancer-related death. Cancer stem cells (CSCs) are regarded as a major factor in determining cancer recurrence, metastasis, and resistance to therapies and are the major obstacle to improving prognosis in cancers, including HCC.^1,2^ Studies on the underlying mechanisms of recurrence, metastasis, and therapeutic resistance are essential for the development of new therapeutic strategies for HCC.

Non-muscle myosin heavy chain IIA (MYH9) is involved in many biological processes, including cell migration, adhesion, division, polarity, and morphogenesis.^3^ MYH9 is dysregulated and serves as an oncogene in many cancers;^4–10^ however, its expression is positively associated with a good prognosis in patients with head and neck squamous cell carcinomas.^11^ Moreover, researchers have reported that MYH9 promotes cell invasion and metastasis in gastric cancer,^12,13^ facilitates cell growth and metastasis in colorectal cancer and pancreatic cancer,^14,15^ and induces drug resistance in neuroblastoma.^16^ However, the role of MYH9 in modulating HCC stemness has yet to be determined.

Chronic hepatitis B virus (HBV) infection has been shown to be a dominant risk factor in most regions of Asia and Sub-Saharan Africa that harbor a high incidence of HCC, including China.^17–19^ The HBV X protein (HBX) has a key role in HBV-induced HCC and has been reported to modulate the expression of numerous genes, as well as epigenetic molecules and events, leading to the deregulation of various pathways and processes in HCC.^20–22^ The Wnt/β-catenin signaling pathway is commonly hyperactivated in CSCs.^23–25^ HBX has been recognized as a critical element of Wnt signaling activation and enhances the stabilization of β-catenin to boost Wnt/β-catenin signaling.^26,27^ However, the mechanism by which HBX promotes Wnt signaling activation has not been fully elucidated.

Here, we describe an MYH9/GSK3β/β-catenin/c-Jun regulatory circuit that enhances HCC cell stemness properties, migration, invasion, growth, and sorafenib resistance. Intriguingly, this positive regulatory circuit is modulated by HBX, which degrades the GSK3β protein through TRAF6-induced ubiquitination and further facilitates β-catenin/c-Jun signaling activation. Data from cell lines, xenograft models, and clinical samples comprehensively characterized the mechanism in which targeting MYH9 delays HCC progression. Accordingly, targeting MYH9 may be an alternative strategy for the treatment of HCC.

## Results

### MYH9 enhances cancer stemness properties, metastasis, proliferation, and sorafenib resistance

**Fig. 1:**
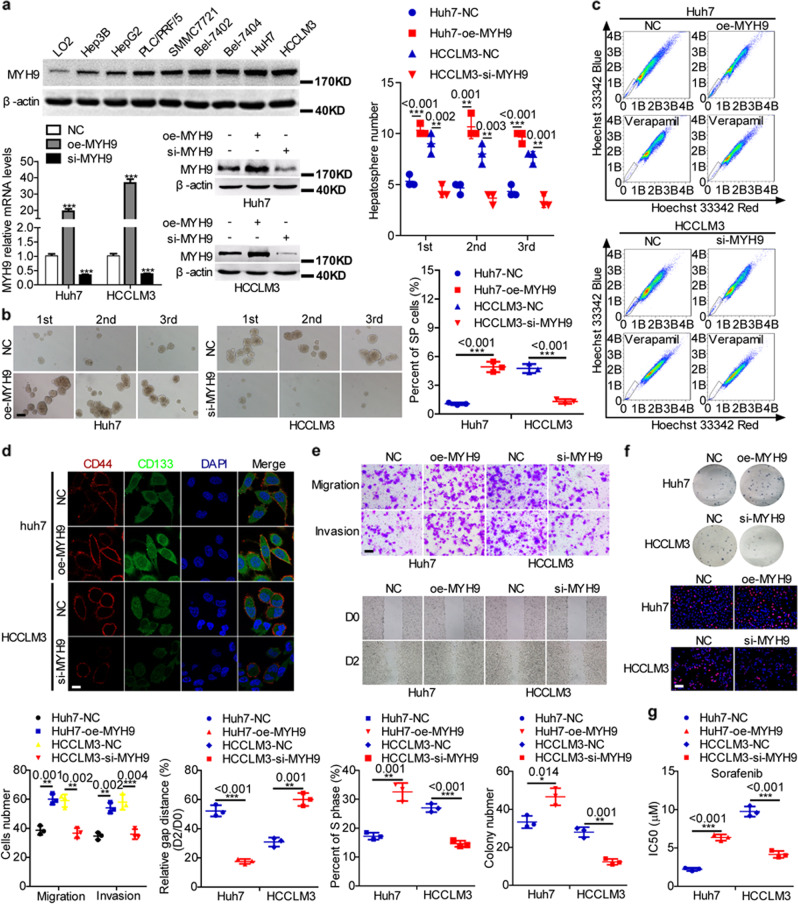
MYH9 promotes stemness properties, migration, invasion, growth, and sorafenib resistance in HCC. **a** Western blotting analyses of MYH9 expression in Hep3B, HepG2, PLC/PRF/5, SMMC-7721, Bel-7402, Bel-7404, Huh7, HCCLM3, and LO2 cells. Western blotting and QPCR analyses of MYH9 levels in MYH9-overexpressing HCC cells, MYH9-depleted HCC cells, and control cells. **b**–**f** Hepatosphere formation assays (scale bar indicates 20 μm) **b**, flow cytometry analyses **c**, immunofluorescence analyses (scale bar indicates 5 μm) **d**, wound healing assays and transwell assays (scale bar indicates 10 μm) **e**, EdU incorporation assays (scale bar indicates 10 μm) and colony-formation assays **f**, and anticancer drug sensitivity tests **g** in MYH9-overexpressing Huh7 cells, MYH9-depleted HCCLM3 cells, and control cells.

To elucidate the role of MYH9 in HCC, we detected MYH9 expression in HCC cells and introduced a plasmid or siRNA to MYH9 into HCC cells and showed a more than twofold change in MYH9 levels in HCC cells treated with the MYH9 plasmid or siRNA compared with the corresponding control cells (Fig. 1a). Silencing MYH9 inhibited HCC stemness, metastasis, proliferation, and sorafenib resistance. However, the upregulation of MYH9 in HCC cells exerted the opposite effects (Fig. 1b–g).

**Fig. 2:**
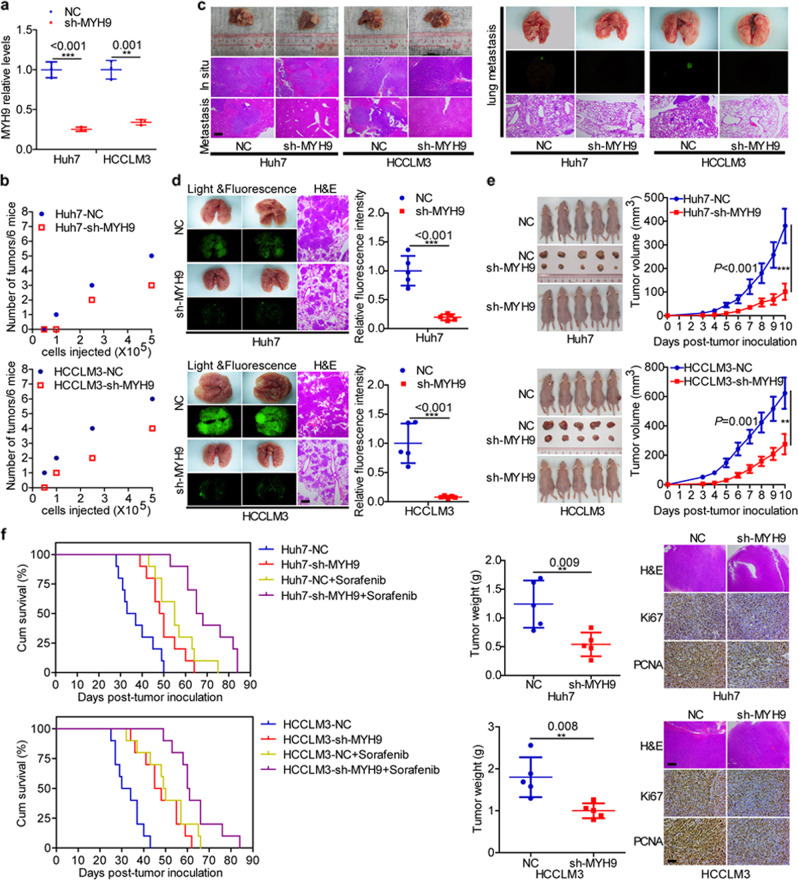
MYH9 enhances the stemness properties, migration, invasion, growth, and sorafenib resistance of HCC cells in vivo. **a** QPCR analyses of MYH9 levels in xenografts originating from Huh7 and HCCLM3 cells with stable silencing of MYH9 and corresponding control cells. **b** A subcutaneous xenograft mouse model was established to elucidate the impact of MYH9 on the tumor-initiating frequency (*n* = 6 per group). **c** The orthotopic tumor model was used to elucidate the effect of MYH9 on metastasis and proliferation (*n* = 7 per group). **d** A pulmonary metastasis model was generated to investigate the impact of MYH9 on metastasis (*n* = 5 per group). **e** A subcutaneous xenograft mouse model was used to elucidate the function of MYH9 on proliferation (general linear model, *n* = 5 per group). Xenografts were stained with H&E and subjected to immunohistochemistry for Ki67 and PCNA expression (*n* = 5 per group). **f** Survival analyses showing the overall survival of HCC-bearing mice in the indicated groups (log-rank test, *n* = 10 per group). Scale bars indicate 50 μm for H&E staining. Scale bars indicate 10 μm for immunohistochemistry.

Mouse models were then established to investigate the in vivo oncogenic role of MYH9. QPCR confirmed the reduced MYH9 expression in tumors derived from MYH9-silenced HCC cells compared with control tumors (Fig. 2a). Subsequently, mice injected with MYH9-silenced HCC cells presented decreased tumor formation ability compared with the control HCC cells in an established subcutaneous xenograft mouse model (Fig. 2b). In a constructed in situ hepatic cancer model, MYH9-silenced Huh7 and HCCLM3 cells displayed a decelerated tumor growth rate, intrahepatic metastasis, and extrahepatic dissemination in nude mice compared with control cells (Fig. 2c). Compared with the metastatic lesions in the corresponding control group, fewer metastatic lesions were measured in animals inoculated with MYH9-silenced Huh7 and HCCLM3 cells. The results were verified by fluorescence and histopathologic changes in the pulmonary metastasis model (Fig. 2d). Compared with the control group, the subcutaneous xenograft mouse model results showed that animals injected with MYH9-silenced cells displayed reduced tumor burden and reduced Ki67 and PCNA expression levels (Fig. 2e). In addition, we observed a prolonged survival of HCC-bearing mice in the sorafenib-treated group and MYH9-silenced group compared with the controls. Interestingly, combining sorafenib therapy and MYH9 knockdown delayed HCC progression (Fig. 2f).

### MYH9 interacts with GSK3β and favors cancer stemness properties, metastasis, proliferation, and sorafenib resistance

**Fig. 3:**
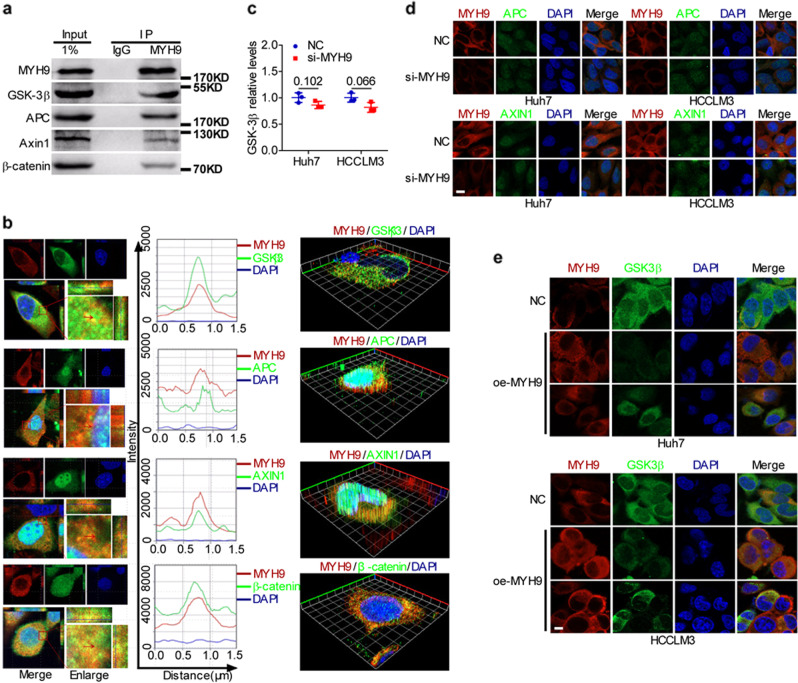
MYH9 interacts with GSK3β to suppress its expression. **a** Coimmunoprecipitation analyses of the interaction between MYH9 and the β-catenin destruction complex in HCCLM3 cells. **b** Immunofluorescence costaining of MYH9 and the β-catenin destruction complex to indicate the colocalization in HCCLM3 (left) and Huh7 (right) cells. The colocalization of MYH9 and the β-catenin destruction complex is shown by calculating the fluorescence intensities along the red arrow crossing the cytoplasm. **c** QPCR analyses of GSK3β mRNA levels in MYH9-depleted Huh7 and HCCLM3 cells and control cells. **d** Immunofluorescence staining of MYH9 and APC or AXIN1 expression and localization in MYH9-silenced Huh7 and HCCLM3 cells and control cells (scale bar indicates 5 μm). **e** MYH9 and GSK3β expression and localization were detected by immunofluorescence costaining in MYH9-overexpressing Huh7 and HCCLM3 cells and control cells (scale bar indicates 5 μm).

To further explore the mechanism of MYH9 in HCC, we focused on the protein interacting with MYH9. Here, we predicted an interaction between the myosin head motor domain, myosin N-terminal SH3-like domain, and RGS domain of MYH9 and the protein kinase domain of GSK3β-based on the DOMINE data sets (http://domine.utdallas.edu/).^28^ Moreover, in the MYH9-associated co-IP complex, we detected the main components of the β-catenin destruction complex, including GSK3β, APC, AXIN1, and β-catenin (Fig. 3a). We also detected the colocalization of MYH9 and the β-catenin destruction complex by immunofluorescence (Fig. 3b). However, MYH9 knockdown had no effect on GSK3β mRNA levels (Fig. 3c). In addition, immunofluorescence indicated that MYH9 reduced the cytoplasmic enrichment of GSK3β but not that of APC and AXIN1, and MYH9 had no effect on the subcellular localization of GSK3β, APC, and AXIN1 (Fig. 3d, e), indicating that MYH9 downregulated GSK3β protein expression at the posttranscriptional level.

Next, we confirmed that MYH9 downregulated the GSK3β protein levels (Supplementary Fig. 1a). In addition to being phosphorylated, GSK3β could also be ubiquitinated and degraded.^29,30^ We showed that MYH9 shortened the half-life of the GSK3β protein (Supplementary Fig. 1b). MYH9 knockdown suppressed GSK3β ubiquitination (Supplementary Fig. 1c). Furthermore, knockdown of GSK3β abolished the inhibitory effects of MYH9 depletion on HCC stemness, migration, invasion, proliferation, and sorafenib resistance (Supplementary Fig. 1d–g). To verify the tumor-suppressive function of GSK3β, we performed TOP/FOP luciferase reporter assays and found that silencing GSK3β antagonized the suppressive effects of MYH9 knockdown on Wnt signaling (Supplementary Fig. 1h). In addition, western blotting data showed that MYH9 knockdown induced E-cadherin and GSK3β expression levels and suppressed the expression levels of Vimentin, N-cadherin, and the targets of Wnt signaling, including β-catenin, c-Jun, c-Myc, CD44, Nanog, Oct4, Sox2, Slug, CCND1, ABCB1, and ABCG2. GSK3β knockdown in MYH9-silenced HCC cells restored the changes in expression signatures regulated by MYH9 knockdown (Supplementary Fig. 1i). Our data demonstrate that MYH9 potentiates HCC progression and Wnt signaling by facilitating GSK3β ubiquitination and degradation.

### HBX interacts with MYH9 and induces its expression to facilitate ubiquitin-mediated GSK3β degradation

**Fig. 4:**
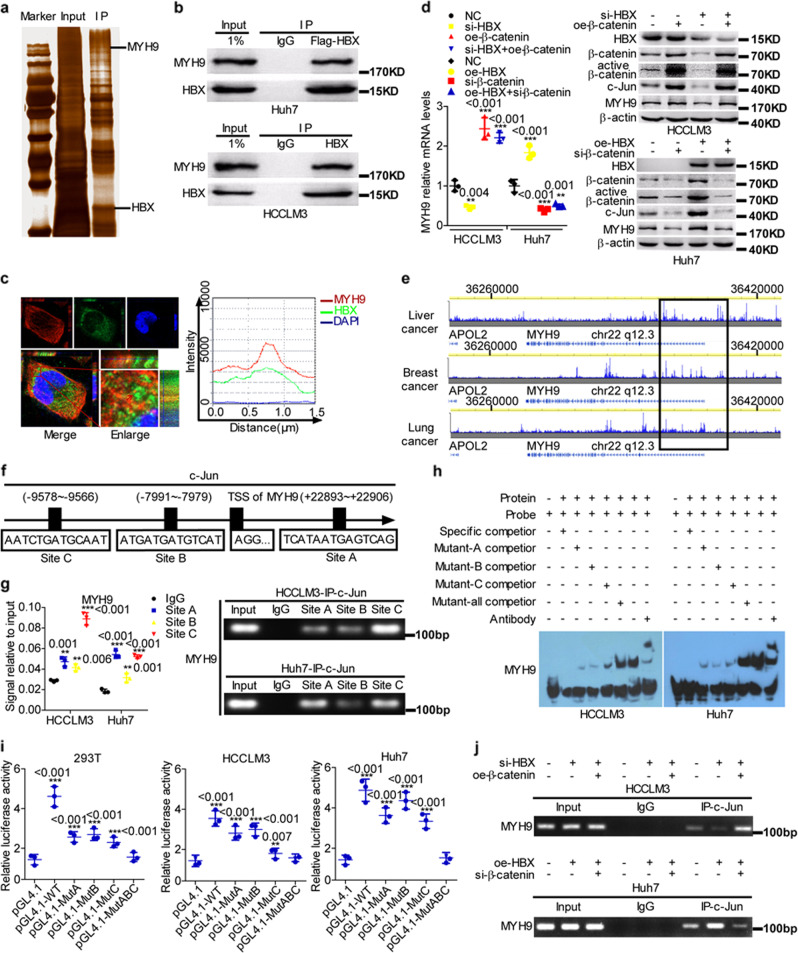
HBX protein interacts with MYH9 and induces its expression. **a** Coimmunoprecipitation and silver staining analyses of HBX-associated proteins in HCC cells. **b** Coimmunoprecipitation analyses of the interaction between HBX and MYH9 in HBX-overexpressing Huh7 and HCCLM3 cells. **c** The colocalization of HBX and MYH9 was evaluated by immunofluorescence costaining. The colocalization of HBX and MYH9 was shown by calculating the fluorescence intensities along the red arrow crossing the cytoplasm. **d** QPCR and western blotting analyses were utilized to measure MYH9 expression levels in HCC cells. **e** ChIP-seq binding peaks searched using the Cistrome Data Browser. **f** Bioinformatics tools were adopted to identify c-Jun-binding sites inside the transcriptional regulatory region of MYH9. Chromatin immunoprecipitation analyses **g**, electrophoretic mobility shift assays **h**, and luciferase reporter assays **i** were conducted to elucidate c-Jun binding to the transcriptional regulatory sequences of MYH9. **j** Chromatin immunoprecipitation analyses were conducted to assess c-Jun binding to the transcriptional regulatory sequences of MYH9 in HCC cells.

Previous research has shown that HBX interacts with APC to activate Wnt signaling.^31^ We conducted co-IP combined with mass spectrometry-based quantitative proteomics and identified MYH9 as a novel HBX-associated protein (Fig. 4a–c and Supplementary Table 1).

We then determined that MYH9 expression was regulated by HBX through β-catenin signaling at the transcriptional level (Fig. 4d). Based on the Cistrome Data Browser combined with bioinformatics tools, three c-Jun-binding sites were found within the transcription regulatory sequences of MYH9 (Fig. 4e, f). The interplay between the transcription factor c-Jun and the transcription regulatory sequences of MYH9 was validated by Chromatin immunoprecipitation (ChIP), electrophoretic mobility shift assay (EMSA), and luciferase reporter assays (Fig. 4g–i). Moreover, ChIP data indicated that HBX modulated MYH9 expression through c-Jun-dependent transcription in HCC cells (Fig. 4j).

**Fig. 5:**
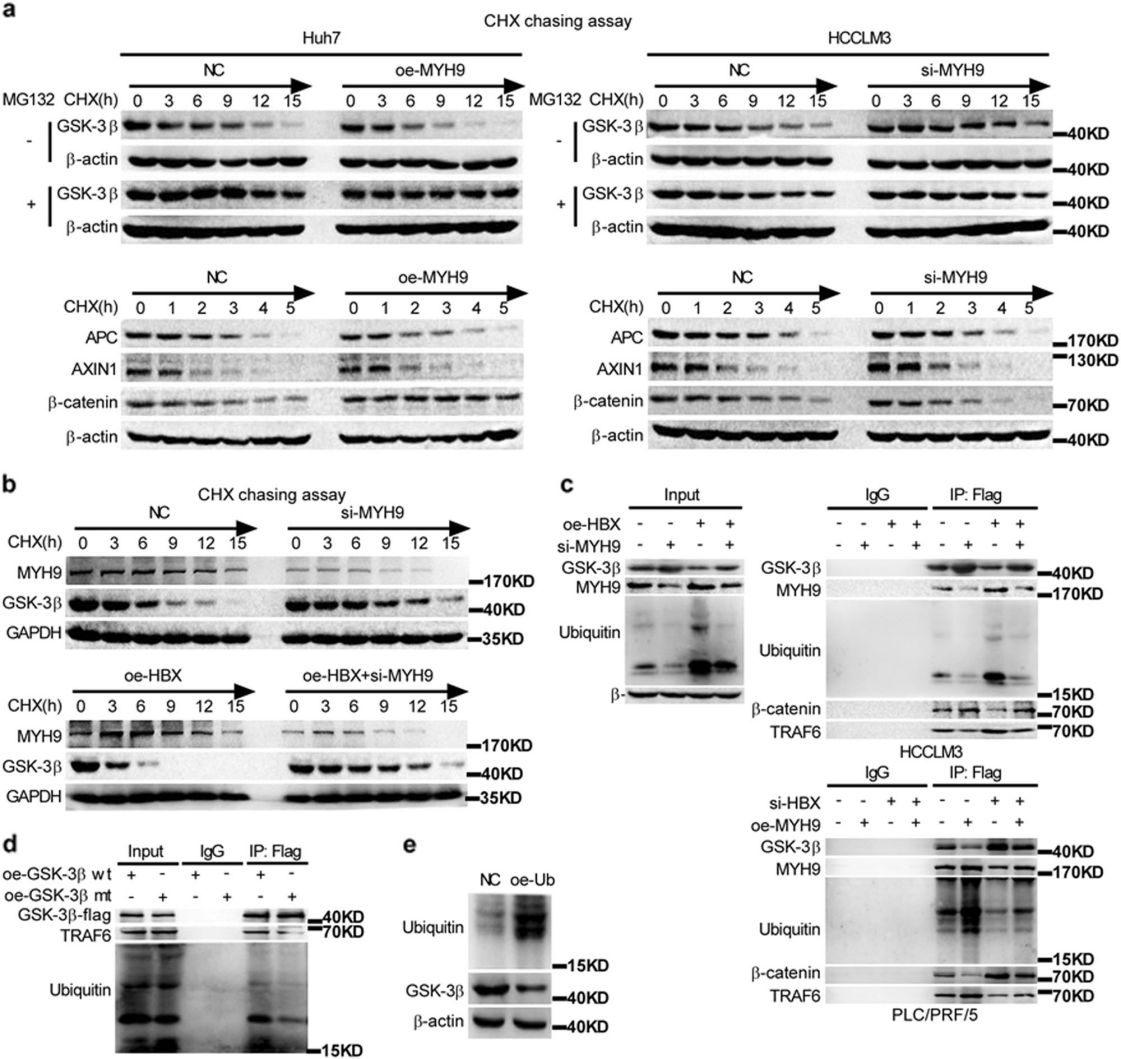
MYH9 mediates HBX-induced GSK3β ubiquitination and degradation. **a** Western blotting and quantification analyses of the effect of MYH9 depletion on GSK3β, β-catenin, APC, and AXIN1 stability in HCCLM3 cells incubated with cycloheximide or MG132 at the indicated time points. **b** Western blotting and quantification analyses of the impact of HBX and MYH9 on GSK3β stability in HCCLM3 cells incubated with cycloheximide at the indicated time points. **c** Coimmunoprecipitation analyses were conducted to identify the function of HBX and MYH9 on the interplay between MYH9, TRAF6, ubiquitin, β-catenin, and GSK3β in HCCLM3 and PLC/PRF/5 cells incubated with MG132. **d** Coimmunoprecipitation analyses of the interaction between TRAF6, ubiquitin, and wild-type GSK3β or mutant GSK3β in HCCLM3 cells treated with MG132. **e** Western blot analyses were used to assess the impact of ubiquitin on GSK3β expression levels in ubiquitin-overexpressing HCCLM3 cells and the corresponding control cells.

To investigate the effect of HBX on MYH9-induced GSK3β protein downregulation, we employed a cycloheximide chasing assay. MYH9 accelerated the degradation of GSK3β and promoted the stabilization of β-catenin but not of other components of the β-catenin destruction complex (Fig. 5a). Moreover, HBX affected GSK3β stability through MYH9-mediated GSK3β degradation; however, HBX had no effect on MYH9 stability (Fig. 5b). Co-IP demonstrated that the upregulation of HBX promoted GSK3β degradation, and the depletion of MYH9 ameliorated the effect of HBX on the ubiquitination and degradation of GSK3β. Co-IP further implied that MYH9 or HBX depletion triggered the dissociation of ubiquitin and GSK3β, accelerated the dissociation of the ubiquitin ligase complex composed of TRAF6 and GSK3β, and facilitated the capture of β-catenin by GSK3β. However, the upregulation of MYH9 or HBX produced the opposite results (Fig. 5c). K183 of GSK3β has been recognized as the ubiquitination site,^32^ and exogenous co-IP further suggested that the mutation of GSK3β K183R prompted the disassembly of ubiquitin and GSK3β and impeded the interplay of TRAF6 and GSK3β (Fig. 5d). Moreover, upregulation of ubiquitin impaired GSK3β expression in HCCLM3 cells (Fig. 5e).

### MYH9 induces the expression of ubiquitin

Intriguingly, we discovered that ubiquitin might be transcriptionally regulated by the transcription factor c-Jun based on the Cistrome Data Browser in conjunction with bioinformatics software analysis (Supplementary Fig. 2a). Furthermore, c-Jun mediated the stimulatory effect of MYH9 on ubiquitin expression (Supplementary Fig. 2b). The interactions between the transcription factor c-Jun and the transcription regulatory sequences of ubiquitin were tested by ChIP, EMSA, and luciferase reporter assays (Supplementary Fig. 2c–e). Furthermore, we verified that MYH9 could regulate the c-Jun-dependent transcription of ubiquitin in HCC cells (Supplementary Fig. 2f).

### HBX stimulates cancer stemness properties, metastasis, proliferation and sorafenib resistance via the MYH9-mediated Wnt/β-catenin/c-Jun signaling pathway

To further investigate the roles of MYH9 and HBX in Wnt signaling, we measured pathway activity by TOP/FOP luciferase reporter assays and showed that MYH9 knockdown ameliorated the promoting effects of HBX on Wnt signaling (Supplementary Fig. 3a). Moreover, MYH9 exerted its oncogenic properties by activating Wnt signaling and modulated the oncogenic effects of HBX on HCC cell stemness, migration, invasion, growth, and sorafenib resistance (Supplementary Fig. 3b–i and Supplementary Fig. 4). In addition, MYH9 controlled the stimulatory effect of HBX on MYH9 and ubiquitin protein levels and the inhibitory effect of HBX on total GSK3β protein levels (Supplementary Fig. 3j).

We further determined the effect of HBX and MYH9 on β-catenin nuclear translocation. Immunofluorescence and cell fractionation assays indicated that MYH9 modulated the promoting effect of HBX on β-catenin nuclear translocation; however, HBX had no effect on the subcellular localization of MYH9 (Supplementary Fig. 3k, l). Furthermore, MYH9 could regulate the augmentative effects of HBX on the c-Jun-dependent transcription of MYH9 and ubiquitin in Huh7 and HCCLM3 cells (Supplementary Fig. 3m). Our results indicate that MYH9 is essential for HBX-mediated Wnt and c-Jun signaling activation in HCC.

To ensure the function of GSK3β and HBX in Wnt signaling, we performed TOP/FOP luciferase reporter assays and found that silencing GSK3β antagonized the suppressive effects of HBX knockdown on Wnt signaling (Supplementary Fig. 5a). However, HBX had no effect on GSK3β mRNA levels (Supplementary Fig. 5b). Furthermore, knockdown of GSK3β abolished the inhibitory effects of HBX depletion on HCC stemness, migration, invasion, growth, and sorafenib resistance (Supplementary Fig. 5c–f). Western blotting data indicated that HBX knockdown induced E-cadherin and GSK3β expression levels, and suppressed the expression levels of Vimentin, N-cadherin, and the targets of Wnt signals, including β-catenin, c-Jun, c-Myc, CD44, Nanog, Oct4, Sox2, Slug, CCND1, ABCB1, and ABCG2. In addition, the downregulation of GSK3β in HBX-silenced HCC cells restored the expression pattern affected by HBX knockdown (Supplementary Fig. 5g). Our data demonstrate that HBX potentiates Wnt signaling by facilitating GSK3β ubiquitination and degradation.

Collectively, our data demonstrate that HBX potentiates Wnt signaling by facilitating MYH9-mediated ubiquitination of GSK3β and promotes HCC progression.

### Correlations between MYH9, GSK3β, and β-catenin levels and clinicopathological features in HCC

Based on TCGA database and bioinformatics analyses, MYH9, β-catenin, and ubiquitin expression levels were upregulated in HCC tissues (T) compared to para-carcinoma tissues (N) (Supplementary Fig. 6a). Intriguingly, MYH9 expression levels were upregulated in HBV-positive compared to HBV-negative HCC samples (Supplementary Fig. 6b). Furthermore, MYH9 was shown to participate in the modulation of the Wnt/β-catenin signaling pathway, cell cycle progression, and focal adhesions by gene set enrichment analysis (GSEA) (Supplementary Fig. 6c). In addition, MYH9 expression was positively correlated with Sox2, Oct4, Epcam, CD44, CD133, β-catenin, N-ca, Vimentin, Slug, CCND1, ABCB1, and ubiquitin expression, and c-Jun expression was positively correlated with MYH9 and ubiquitin expression (Supplementary Fig. 6d). According to the best cutoff value of MYH9 expression, we assigned HCC patients into two groups and found that MYH9 expression levels were positively associated with poor patient prognosis based on TCGA database analysis (Supplementary Fig. 6e).

**Fig. 6:**
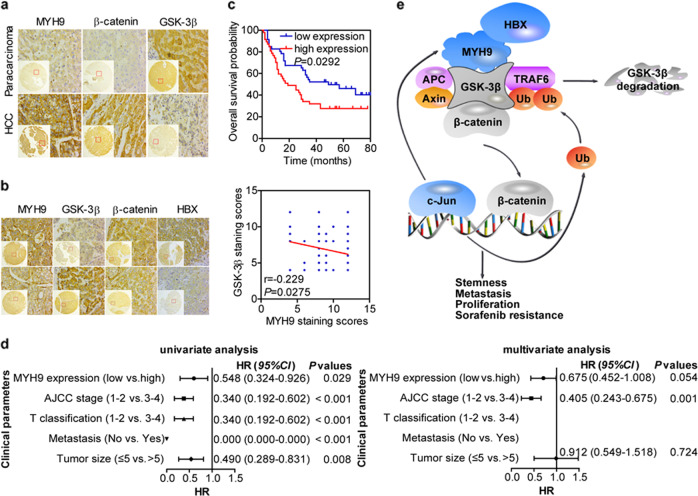
Immunohistochemical analyses of MYH9, GSK3β, β-catenin, and HBX expression in HCC. **a** Comparisons of MYH9, GSK3β, and β-catenin expression between HCC and adjacent noncancerous tissues. **b** Correlations among HBX, MYH9, GSK3β, and β-catenin expression. **c** Kaplan–Meier survival analysis was performed for HCC patients according to MYH9 expression. **d** Univariate and multivariate survival analyses of clinicopathological characteristics of hepatocellular carcinoma patients. **e** Working model of the MYH9/GSK3β/β-catenin-c-Jun regulatory circuit activated by HBX via MYH9-modulated ubiquitination and degradation of GSK3β in Wnt signaling in HCC.

Immunohistochemistry (IHC) was then performed in 93 HCC tissues and 87 adjacent noncancerous tissues. Consistent with the previous findings, MYH9 and β-catenin expression levels were elevated (*P* < 0.001 and *P* = 0.007, respectively), but GSK3β expression levels were decreased in HCC tissues in contrast to those in para-carcinoma tissues (*P* < 0.001) (Fig. 6a). In addition, MYH9 expression levels were negatively associated with GSK3β expression levels and positively associated with HBX expression levels (*P* = 0.012, *Kappa* = 0.254) and β-catenin nuclear translocation (*P* = 0.003, *Kappa* = 0.309) (Fig. 6b and Supplementary Tables 2, 3).

Moreover, survival analysis illustrated that MYH9 expression levels were positively associated with poor patient prognosis (median survival of 19 months for the high MYH9 group versus 49 months for the low MYH9 group) (log-rank test, *P* = 0.0292) (Fig. 6c).^33^ Importantly, univariate and multivariate analyses suggested that MYH9 expression was an independent indicator for the overall survival of HCC patients (Fig. 6d).

## Discussion

In this study, we demonstrated that MYH9 was responsible for HBX-induced HCC progression. MYH9 activated Wnt/β-catenin and its downstream oncogenic signaling pathways. This effect promoted the CSC properties, migration, invasion, proliferation, and sorafenib resistance of HCC cells. Specifically, inhibition of MYH9 prevented HCC progression, indicating that MYH9 can be applied as an alternative therapeutic target for HCC.

MYH9 has been shown to play dual functions in cancers. In the present work, we first confirmed the oncogenic role of MYH9 in HCC in vitro and in vivo. Mechanistic analysis identified Wnt/β-catenin and its mediated tumor stemness, EMT and c-Jun as positive downstream signaling pathways of MYH9 in HCC development. In addition, we elucidated the upstream stimulatory factor in the regulation of MYH9 expression. The transcription factor c-Jun is a downstream driver of the Wnt/β-catenin signaling pathway.^34^ Interestingly, we further determined that c-Jun transcriptionally activated MYH9 and ubiquitin expression to participate in MYH9-mediated ubiquitination and degradation of GSK3β, thus forming the positive regulatory loop MYH9/GSK3β/β-catenin/c-Jun in HCC progression. Here, our data demonstrated the significance of MYH9 in HCC pathogenesis.

The oncogenic activity of HBX has been well characterized in HCC.^35–38^ Consistent with previous reports,^39,40^ HBX contributed to hepatocarcinogenesis in this study by promoting stemness, metastasis, proliferation, and sorafenib resistance. In the present work, we identified a new HBX-associated protein, MYH9, which interacted with the β-catenin degradation complex consisting of GSK3β, APC, and AXIN1. HBX potentiated the Wnt/β-catenin and c-Jun signaling pathways via MYH9-mediated ubiquitination and degradation of GSK3β, providing a new mechanism by which HBX modulated the expression of proteins associated with stemness, metastasis, proliferation, and sorafenib resistance. In addition to the phosphorylation of GSK3β, we also found that GSK3β could be ubiquitinated in HCC. A previous study suggested that the ubiquitination of GSK3β affected the interaction between GSK3β and TRAF6 and regulated the activity of GSK3β.^32^ However, we demonstrated that GSK3β ubiquitination influenced its stability by binding to the E3 ubiquitin ligase TRAF6, and lysine 183 was essential for GSK3β polyubiquitination, although the half-life of the GSK3β protein exceeded 8 h. According to previous studies,^41,42^ we surmised that MYH9 functioned as a scaffold in a multiprotein complex and recruited the E3 ubiquitin ligase TRAF6 to GSK3β, thus facilitating the interaction between GSK3β and TRAF6. In addition, we verified that HBX potentiated the GSK3β-mediated Wnt/β-catenin/c-Jun signaling pathway, enhancing the CSC properties, migration, invasion, proliferation, and sorafenib resistance of HCC cells. This result is similar to a previous study suggesting that decreased expression of GSK3β is responsible for promoting the activity of Wnt/β-catenin signaling in HCC.^43^ Moreover, these findings suggested that HBX induced the MYH9/GSK3β/β-catenin/c-Jun-positive regulatory circuit to increase the activity of Wnt/β-catenin/c-Jun signaling and promote the CSC properties, migration, invasion, proliferation, and sorafenib resistance of HCC cells.

Bioinformatics and immunohistochemical assays supported the notion that MYH9 was involved in HBV-modulated HCC progression and was positively associated with poor patient prognosis. Correlation analysis of MYH9, GSK3β, β-catenin, ubiquitin, and c-Jun expression further confirmed the results observed in HCC cells. MYH9 was positively correlated with the expression of several genes that contributed to HCC cell stemness, metastasis, proliferation, and sorafenib resistance, and c-Jun was positively correlated with MYH9 and ubiquitin expression. Additionally, GSEA verified that MYH9 modulated the Wnt/β-catenin signaling pathway and HCC progression. Taken together, these findings reveal that HBX/MYH9/GSK3β/β-catenin, ubiquitin, and c-Jun play a crucial role in HCC progression.

In summary, this work demonstrates the dominant function of MYH9 in inducing cancer stemness and progression of HCC. Targeting MYH9 remarkably improved the survival of mice and facilitated HCC cell treatment sensitivity in vivo. In addition, our study delineates a new mechanism by which HBX induces the MYH9/GSK3β/β-catenin/c-Jun regulatory circuit, which synergistically fosters HCC development by permitting the autonomous regulation of the Wnt/β-catenin signaling pathway (Fig. 6e). Specifically, bioinformatics analysis and our patient data strengthened the significance and reliability of the results on MYH9 and its related pathway. Our findings contribute to the understanding of the underlying role of MYH9 in regulatory networks and emphasize the biological and clinical basis for the potential application of MYH9 as a new indicator of HCC diagnosis and an effective target for HCC treatment.

## Materials and methods

### Cell culture

Hep3B, HepG2, PLC/PRF/5, SMMC-7721, Bel-7402, Bel-7404, Huh7, and HCCLM3 cells were obtained from the Cell Bank of the Chinese Academy of Sciences. HEK293T and LO2 cells were obtained from the Cancer Research Institute of Southern Medical University. Cells were cultured in Dulbecco's Modified Eagle Medium (DMEM) containing fetal bovine serum (10%) (Biowest, Loire Valley, France) at 37 °C with 5% CO_2_ in an incubator.

### Cell transfection

siRNAs were designed, synthesized, and obtained from RiboBio Corporation (Guangzhou, China) (Supplementary Table 4). Plasmids were obtained from Vigene Biosciences Corporation (Shandong, China). Before transfection, cells were plated in a cell culture dish or plate. The transfection of plasmids and/or siRNAs was performed by Lipofectamine 2000. Forty-eight to 72 h later, the cells were collected for further experiments.

### Lentivirus production and infection

The establishment of lentiviral vectors harboring shRNA-targeting MYH9 and the construction of Huh7 and HCCLM3 cell lines stably expressing shRNA were performed as previously described.^44^ In brief, the MYH9 shRNA sequence was selected from the NM_002473.5 MYH9 mRNA sequence (Supplementary Table 4) by the BLOCK-It RNAi Designer (Thermo Fisher Scientific). The lentiviral vector containing human shRNA-MYH9 was prepared by the pLVTHM-GFP lentiviral RNAi expression system. HCC cells were transfected with lentiviral particles harboring experimental or control vectors, and the expression of MYH9 was measured by QPCR.

### QPCR and RT-PCR

Total RNA was harvested, and cDNA was generated by a reverse transcription reagent kit purchased from TaKaRa company. Then, the cDNA template was used for amplification with specific primers (Supplementary Table 5). The Bio-Rad CFX 96 and Bio-Rad T100 detection systems were used for QPCR and RT-PCR, respectively.

### Western blot analysis

Proteins were obtained from cell lysates in lysis buffer and were used for protein quantification. Proteins were separated by sodium dodecyl sulfate polyacrylamide gel electrophoresis, transferred to polyvinylidene difluoride membranes, and subjected to immunoprobing with specific antibodies. A chemiluminescence reagent kit purchased from Thermo Fisher Scientific company was used to detect the proteins. Antibodies against the following antigens were used: HBX, MYH9, GSK3β, APC, AXIN1, TRAF6, ubiquitin, active β-catenin, β-catenin, c-Jun, CD44, c-Myc, N-cadherin, E-cadherin, vimentin, Slug, Nanog, OCT4, SOX2, ABCG2, ABCB1, CCND1, and β-actin. Images were obtained by a Bio-Rad ChemiDoc CRS+Molecular Imager.

### Tumorsphere formation assay

Cells (5 × 10^3^) were dissociated into single-cell suspensions and plated in six-well ultra-low-attachment plates. The seeded cells were propagated in serum-free DMEM/F12 with 20 ng/ml epidermal growth factor, 20 ng/ml fibroblast growth factors, and 2% B27. The growth of tumorspheres was measured using a microscope after incubation for 2–3 weeks, and the tumorspheres were then dissociated into single cells to generate new tumorspheres. Tumorspheres were passaged for three generations, and the number and size of tumorspheres were calculated for further analysis.

### Flow cytometry analysis

For analysis of the proportion of CD44^high^CD133^high^ cells, cells (1 × 10^6^) were dissociated into single-cell suspensions and were then resuspended in 200 µl of phosphate-buffered saline (PBS) and incubated with phycoerythrin (APC)-conjugated anti-CD133 and fluorescein isothiocyanate-conjugated anti-CD44 antibodies. The cells were incubated for 30 min on ice, washed with PBS, and subjected to flow cytometry analysis. For side population analysis, cells (1 × 10^6^) were dissociated into single-cell suspensions in DMEM supplemented with 2% fetal bovine serum (FBS) and were then incubated with Hoechst 33342 (5 μg/ml) purchased from Sigma-Aldrich company with gentle resuspending every 10 min at 37 °C for 90 min. In the negative control group, samples were simultaneously treated with verapamil (50 μmol/L) purchased from Sigma-Aldrich company. Cells were rinsed and analyzed by flow cytometry. Propidium iodide staining was applied to detect dead cells.

### Migration, invasion, and wound healing assays

Cell migration and invasion were determined using a Transwell assay. Cells were resuspended in serum-free DMEM and plated in the upper chamber of Transwell inserts coated with or without BD Matrigel, and the lower chambers were filled with DMEM containing FBS (10%). The migrated cells were stained, imaged, and analyzed by calculating the cell numbers from five random fields. Cells were plated and grown into a confluent monolayer in six-well plates. Scratches were then generated using a pipette tip. After wounding, the cell migration process was visualized using a microscope at 0, 24, 48, and 72 h.

### MTT assay

Drug sensitivity tests and cell proliferation were measured by the MTT assay. Cells were plated into 96-well plates. After cell adherence, the cells were treated with drugs or left untreated, and cell viability was detected by the MTT reagent (5 mg/ml) purchased from Sigma-Aldrich company.

### Colony-formation assay

Cells at a density of 200 cells/well were plated in six-well plates. After culturing for 14 days, the generated colonies were fixed with methanol for 15 min, stained with a hematoxylin solution and photographed using a microscope.

### EdU incorporation assay

An Apollo567 in vitro Imaging Kit was purchased from RiboBio Corporation (Guangzhou, China) and was applied for the EdU incorporation assay. After culturing with EdU (10 μm) for 2 h, the cells were fixed with paraformaldehyde (4%), permeabilized with Triton X-100 (0.2%), and costained with 4′,6-diamidino-2-phenylindole (DAPI, 5 μg/ml) and Apollo fluorescent dyes.

### Immunofluorescence and confocal microscopy

Cells were separated and seeded on coverslips in a 48-well plate. After cell adherence, the cells were fixed with paraformaldehyde (4%) and permeabilized with Triton X-100 (0.2%). The cells were then incubated with specific antibodies, counterstained with DAPI (0.2 mg/ml), and imaged under a Carl Zeiss LSM800 confocal laser scanning microscope.

### In vivo tumor xenograft study

The protocols for the mouse experiments conformed to international regulations for animal care and maintenance and were approved by the Institutional Animal Ethical Committee, Experimental Animal Center of Southern Medical University. To evaluate tumor growth, a subcutaneous xenograft mouse model was established. Cells were resuspended in PBS and subcutaneously injected into the flanks of 4- to 5-week-old male BALB/c-nu mice (*N* = 5 per group). Tumors from HCC-bearing mice were excised, weighed, and subjected to further study at 15 days after cell inoculation. Tumor volumes were calculated as previously described.^45,46^ A subcutaneous xenograft mouse model was also adopted to investigate the tumor formation ability as previously described.^43^ In brief, a series of cells were inoculated into the animals (*N* = 6 per group), and tumor formation was measured after cell inoculation. In addition, a subcutaneous xenograft mouse model was also applied to determine HCC resistance to sorafenib. Male BALB/c-nu mice aged 4–5 weeks (*N* = 10 per group) were subcutaneously injected with 5 × 10^6^ cells in the flanks and then treated orally with the vehicle solution or sorafenib (30 mg/kg) once daily for 4 weeks.^47^ Survival curves were generated by Kaplan–Meier analysis.

In vivo metastasis assays were performed using a pulmonary metastasis model and an orthotopic tumor model. Cells were intravenously inoculated into the tail veins of the animals (*N* = 5 per group) or inoculated under the liver capsule (*N* = 7 per group). The metastases were measured as previously described.^48^ Optical and pathological images were obtained to evaluate the growth of primary tumors and the formation of metastatic lesions.

### CHX chase assay

Cells were treated with scramble control, siRNA, or plasmid, and cultured with MG132 (20 μmol/L) or left untreated for 6 h. After treatment with CHX (50 µg/ml), cells were collected at the indicated time points and prepared for western blot analysis.

### Cell fractionation assay

A NE-PER Nuclear and Cytoplasmic Extraction kit purchased from Thermo Fisher Scientific company was used for a cell fractionation assay. Cells were collected, rinsed, and then cultured for 10 min in ice-cold CER I. Then, the reaction mixture was incubated with CER II extraction reagent for an additional 1 min. After centrifugation for 5 min at 16000 × *g*, the cytoplasmic extract in the- supernatant was carefully aspirated and stored on ice. NER extraction reagent was added to the resuspended pellet for incubation for 40 min on ice. After centrifugation for 10 min at 16000 × *g*, the nuclear extract in the supernatant was aspirated and stored on ice. Proteins were quantified and subjected to western blot analysis.

### Luciferase reporter assays

To investigate the function of the transcription factor c-Jun on the transcriptional activity of ubiquitin and MYH9, sequences expressing wild-type or mutant c-Jun-binding sites were inserted into the luciferase reporter vector. The vectors and the c-Jun plasmid were co-transfected into cells, and the Dual-Luciferase Reporter Assay System purchased from Promega Corporation was used to detect luciferase activity.

### TOP/FOP luciferase reporter assay

The Luciferase Assay System was used to perform transcriptional activity assays. Cells were co-transfected with FOPflash or TOPflash with pRL purchased from Millipore Corporation. After transfection for 24 h, the cells were harvested and subjected to luciferase activity measurement on a BioTek luminometer using the Dual-Luciferase Reporter Assay System purchased from Promega Corporation. The ratio of firefly luciferase activity to Renilla luciferase activity was calculated to evaluate the transcriptional activity of each sample.

### ChIP assay

A ChIP assay kit purchased from Thermo Fisher Scientific company was used to perform chromatin immunoprecipitation assays. In brief, chromatin was cross-linked, isolated, and cut into DNA fragments with micrococcal nuclease. Immunoprecipitation was performed with anti-c-Jun or IgG in the reaction system. The recovered DNA fragments were eluted, purified, and analyzed by QPCR and/or PCR.

### EMSA

An EMSA Kit (BersinBio, Guangzhou, China) was used for the EMSA. In brief, nuclear extracts were collected from cell lysates and used for protein quantification. Biotin-labeled probes and nuclear extracts were then added to the reaction system. A 100-fold excess of unlabeled mutant or wild-type probes (cold competitors) (Supplementary Table 6) or anti-c-Jun were added to the reaction system for competition or supershift assays. Signals were recorded and analyzed after electrophoresis and incubation.

### Coimmunoprecipitation (co-IP)

A Pierce Co-IP kit purchased from Thermo Fisher Scientific company was used to carry out co-IP. Total proteins were harvested from cells and used for protein quantification. Specific antibodies (10 μg) or IgG was added to 5 mg protein for overnight incubation. The recovered proteins were eluted and analyzed by silver staining, mass spectrometry, and western blotting.

### Transcriptomics data analysis

Based on data from the TCGA database, mRNA expression profiles of HCC patients were extracted and analyzed as previously described.^49^ The latest mRNA-Seq data for liver HCC were applied in correlation analysis, differential expression analysis, and survival analysis.

### Tissue specimens

Nighty-three paraffin-embedded HCC specimens and 87 paraffin-embedded adjacent nontumor specimens were included in this study. Clinical materials of the patients were collected, and patients who had been treated with chemotherapy, preoperative radiation, or biotherapy were excluded. All samples had a definite pathological diagnosis. Ethics approval from the local ethics committee and patient consent were obtained.

### IHC

Paraffinized sections of the specimens were deparaffinized, dehydrated, and incubated with citrate buffer for 3 min for antigen retrieval. After blocking endogenous peroxidase activity with 3% H_2_O_2_ and nonspecific antigens with goat serum, the sections were incubated with specific antibodies overnight. Then, the sections were rinsed with PBS, incubated with horseradish peroxidase-conjugated antibody, and visualized by 3,3′ diaminobenzidine tetrahydrochloride substrate. The immunohistochemical staining intensity was evaluated as described previously.^50^ A score >6 was classified as high expression, and a score ≤6 was classified as low expression.

### Statistical analysis

All data were analyzed by SPSS 22.0 (SPSS, Inc. Chicago, IL, USA). Data from at least three independent experiments are expressed as the means ± SDs. The general linear model repeated measures variance analysis was performed to determine significance in MTT assays and tumor growth, one-way analysis of variance was performed to determine significance among multiple groups, and Student’s two-tailed *t* test was performed to determine significance between two groups. The Wilcoxon rank-sum test was used for analyzing nonparametric data. Correlations between gene expression levels were elucidated by Spearman’s rank correlation test or the kappa consistency test. Correlations between clinicopathological characteristics and gene expression levels were investigated by the chi-square test. Correlations between gene expression and overall patient survival were detected by log-rank tests of Kaplan–Meier survival curves. A Cox proportional hazards regression model was used for univariate and multivariate survival analyses. All statistical tests were two-sided, and a *P* value of < 0.05 was considered to be statistically significant. *P < 0.05, **P < 0.01, and ***P < 0.001.

## Supplementary information

the revised Supplementary information

## Data Availability

The data sets generated during and/or analyzed during the current study are available from the corresponding author upon reasonable request.
