# Supplementary material for: Silencing MYH9 blocks HBx-induced GSK3β ubiquitination and degradation to inhibit tumor stemness in hepatocellular carcinoma
Source: Signal Transduct Target Ther. 2020 Feb 14;5:13. doi: 10.1038/s41392-020-0111-4 (PMC7018736; doi:10.1038/s41392-020-0111-4)
Supplement: Supplementary file 1 — the revised Supplementary information [file 41392_2020_111_MOESM1_ESM.doc]

Supplementary Materials for

Silencing MYH9 blocks HBx-induced GSK3β ubiquitination and degradation to inhibit tumor stemness in hepatocellular carcinoma

Xian Lin^1#^, Ai-min Li^1#^, Yong-Hao Li^1#^, Rong-Cheng Luo^1#^, Yu-Jiao Zou^1^, Yi-Yi Liu^1^, Chen Liu^1^, Ying-Ying Xie^1^, Shi Zuo^4^, Zhan Liu^5^, Zhen Liu^1,3#^*, Wei-Yi Fang^1,2^*

Correspondence to: fangweiyi1975@163.com

**This PDF file includes:**

Figures. S1 to S6

Tables S1 to S6

Figure. S1.

**
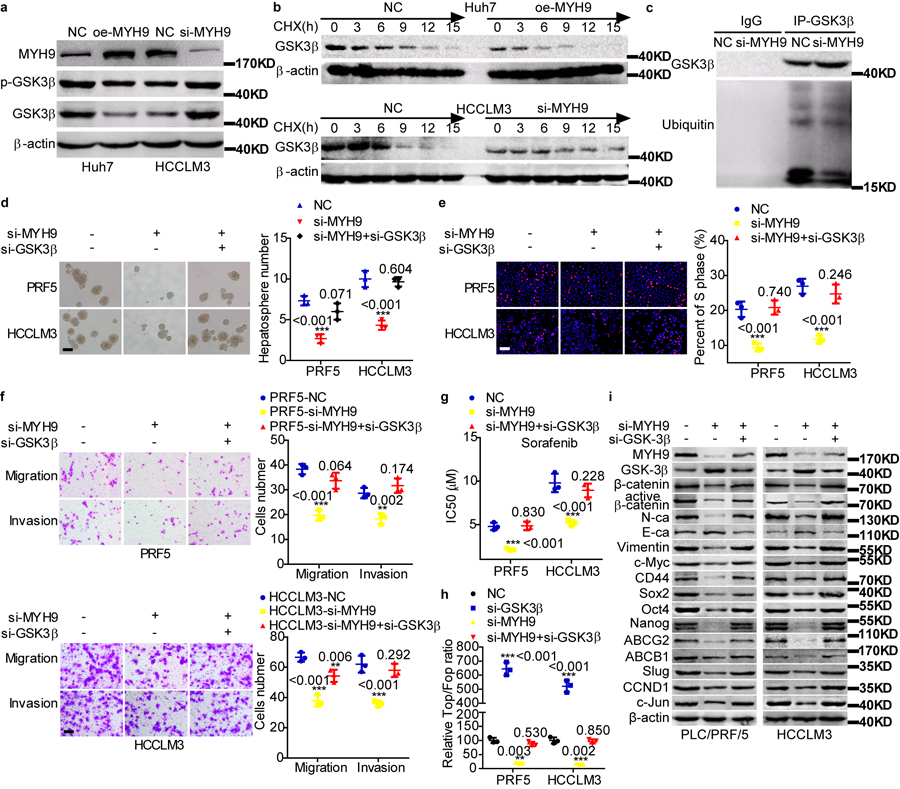
**

**Supplementary Fig. 1** **MYH9 promotes GSK3β-mediated cancer stemness, metastasis, proliferation, and sorafenib resistance in HCC.** (a) Western blotting analyses of GSK3β expression levels in MYH9-overexpressing Huh7, MYH9-depleted HCCLM3, and the control cells. (b) Western blotting and quantification analyses of the impact of MYH9 on GSK3β stability in HCC cells incubated with cycloheximide at indicated time points. (c) Co-immunoprecipitation analyses of the function of MYH9 on the interplay between GSK3β and ubiquitin in HCCLM3 cells incubated with MG132. Hepatosphere formation assays (Scale bar indicates 20 μm) (d), EdU incorporation assays (Scale bar indicates 10 μm) (e), Transwell assays (Scale bar indicates 10 μm) (f), and anti-cancer drug sensitivity tests (g) of GSK3β-depleted HCC cells, MYH9-depleted HCC cells, MYH9-depleted HCC cells with GSK3β depletion, and the control cells. (h) TOP/FOP luciferase reporter assays of Wnt/β-catenin signaling activity in GSK3β-depleted HCC cells, MYH9-depleted HCC cells, MYH9-depleted HCC cells with GSK3β depletion, and the control cells. (i) Western blotting analyses of stemness, metastasis, proliferation, therapy-resistance, and Wnt/β-catenin signaling-associated proteins expression in HCC cells.

Figure. S2.

**
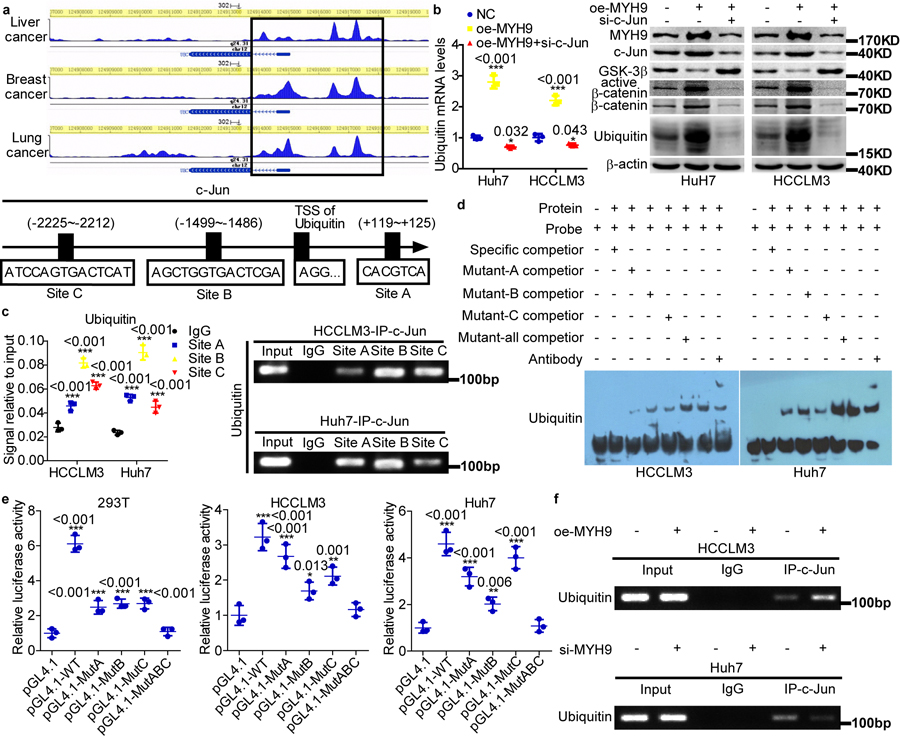
**

**Supplementary Fig. 2** **MYH9 induces ubiquitin expression.** (a) ChIP-seq binding peaks were searched using the Cistrome Data Browser and bioinformatics analyses was applied to identify c-Jun-binding sites inside the transcription regulatory sequences of ubiquitin. (b) QPCR and Western blotting analyses of ubiquitin expression levels in MYH9-overexpressing Huh7 cells, MYH9-overexpressing Huh7 cells with c-Jun knockdown, MYH9-silenced HCCLM3 cells, MYH9-silenced HCCLM3 cells with c-Jun overexpression, and the control cells. Chromatin immunoprecipitation analyses (c), electrophoretic mobility shift assays (d), and luciferase reporter assays (e) of c-Jun binding to the transcriptional regulatory sequences of ubiquitin. (f) Chromatin immunoprecipitation analyses of c-Jun binding to the transcriptional regulatory sequences of ubiquitin in MYH9-overexpressing HCCLM3 cells, MYH9-silenced Huh7 cells, and the control cells.

Figure. S3.


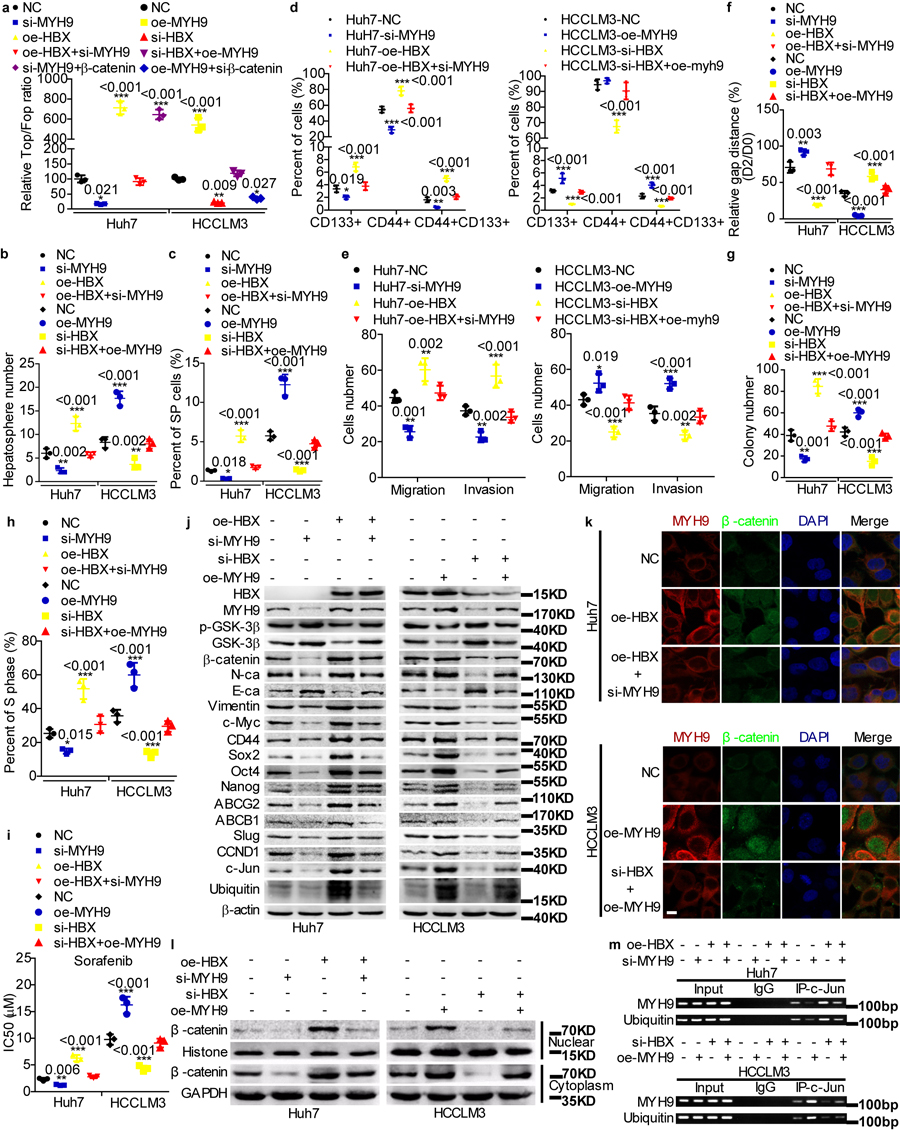


**Supplementary Fig. 3 Hepatitis B viral X protein activates MYH9-mediated Wnt/β-catenin and c-Jun signaling pathways in HCC.** TOP/FOP luciferase reporter assays of Wnt/β-catenin signaling activity (a), and statistical analyses of hepatosphere formation assays (b), flow cytometry analyses (c, d), Transwell assays (e), wound healing assays (f), colony-formation assays (g), EdU incorporation assays (h), and anti-cancer drug sensitivity tests (i) in HCC cells. (j) Western blotting analyses of stemness, metastasis, proliferation, therapy-resistance, and Wnt/β-catenin signaling-associated protein expression in HCC cells. (k) Immunofluorescence costaining of MYH9 and β-catenin expression and localization in HCC cells (Scale bar indicates 5 μm). (l) Nucleic and cytoplasmic β-catenin levels were detected by western blotting analyses in HCC cells. (m) Chromatin immunoprecipitation analyses of c-Jun binding to the transcriptional regulatory sequences of MYH9 and ubiquitin in HCC cells.

Figure. S4.

**
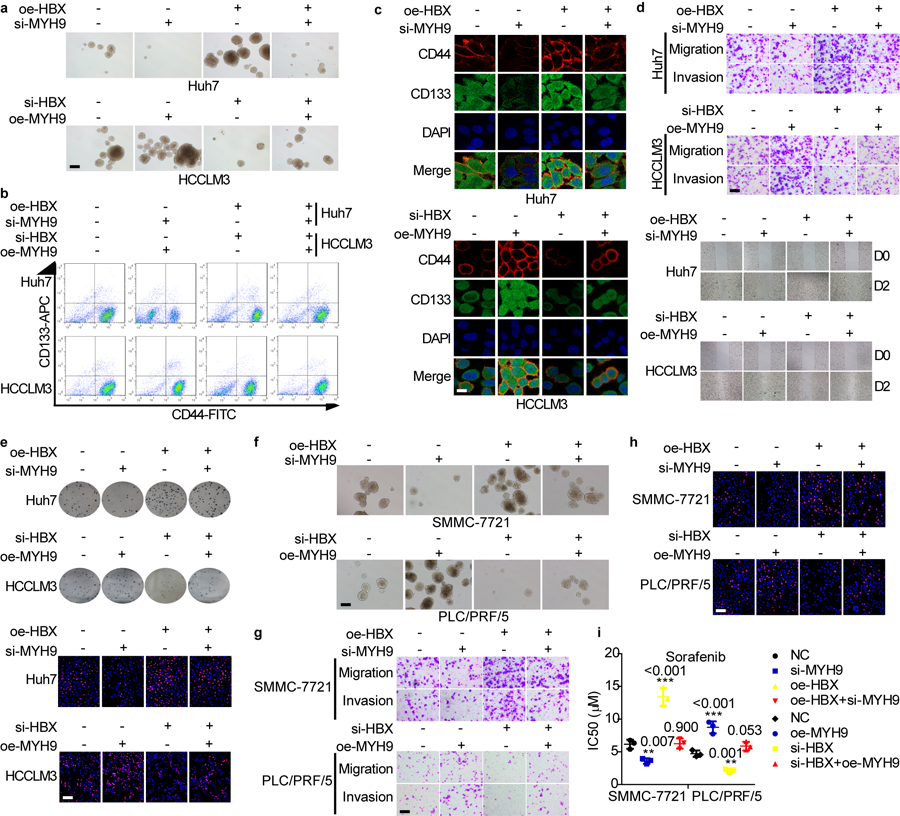
**

**Supplementary Fig. 4** **Hepatitis B viral X protein facilitates MYH9-mediated HCC progression.** Hepatosphere formation assays (Scale bar indicates 20 μm) (a), flow cytometry analyses (b), immunofluorescence analyses (Scale bar indicates 5 μm) (c), wound healing assays and Transwell assays (Scale bar indicates 10 μm) (d), and EdU incorporation assays (Scale bar indicates 10 μm) and colony-formation assays (e) of MYH9-silenced Huh7 cells, HBX-overexpressing Huh7 cells, HBX-overexpressing Huh7 cells with MYH9 knockdown, MYH9-overexpressing HCCLM3 cells, HBX-silenced HCCLM3 cells, HBX-silenced HCCLM3 cells with MYH9 overexpression, and the control cells. Hepatosphere formation assays (Scale bar indicates 20 μm) (f), Transwell assays (Scale bar indicates 10 μm) (g), EdU incorporation assays (Scale bar indicates 10 μm) (h), and anti-cancer drug sensitivity tests (i) of MYH9-silenced SMMC-7721 cells, HBX-overexpressing SMMC-7721 cells, HBX-overexpressing SMMC-7721 cells with MYH9 knockdown, MYH9-overexpressing PLC/PRF/5 cells, HBX-silenced PLC/PRF/5 cells, HBX-silenced PLC/PRF/5 cells with MYH9 overexpression, and the control cells.

Figure. S5.

**
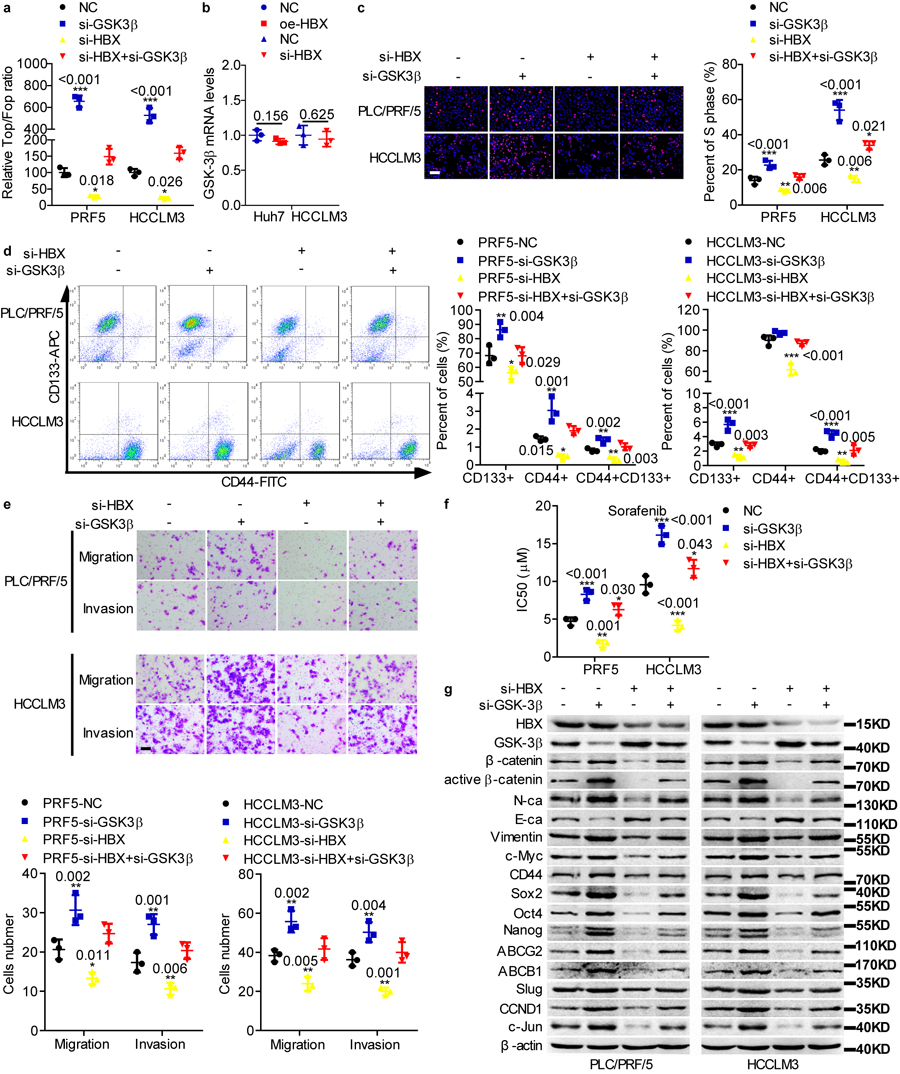
**

**Supplementary Fig. 5** **Hepatitis B viral X protein augments GSK3β-mediated cancer stemness, metastasis, proliferation, chemoresistance and Wnt/β-catenin/c-Jun signaling pathway in HCC.** (a) TOP/FOP luciferase reporter assays of Wnt/β-catenin signaling activity in GSK3β-depleted HCC cells, HBX-depleted HCC cells, HBX-depleted HCC cells with GSK3β depletion, and the control cells. (b) QPCR analyses of GSK3β mRNA levels in HBX-overexpressing Huh7, HBX-depleted HCCLM3 and the control cells. EdU incorporation assays (Scale bar indicates 10 μm) (c), flow cytometry analyses (d), Transwell assays (Scale bar indicates 10 μm) (e), and anti-cancer drug sensitivity tests (f) of GSK3β-depleted HCC cells, HBX-depleted HCC cells, HBX-depleted HCC cells with GSK3β depletion, and the control cells. (g) Western blotting analyses of stemness, metastasis, proliferation, therapy-resistance, and Wnt/β-catenin signaling-associated proteins expression in GSK3β-depleted HCC cells, HBX-depleted HCC cells, HBX-depleted HCC cells with GSK3β depletion, and the control cells.

Figure. S6.


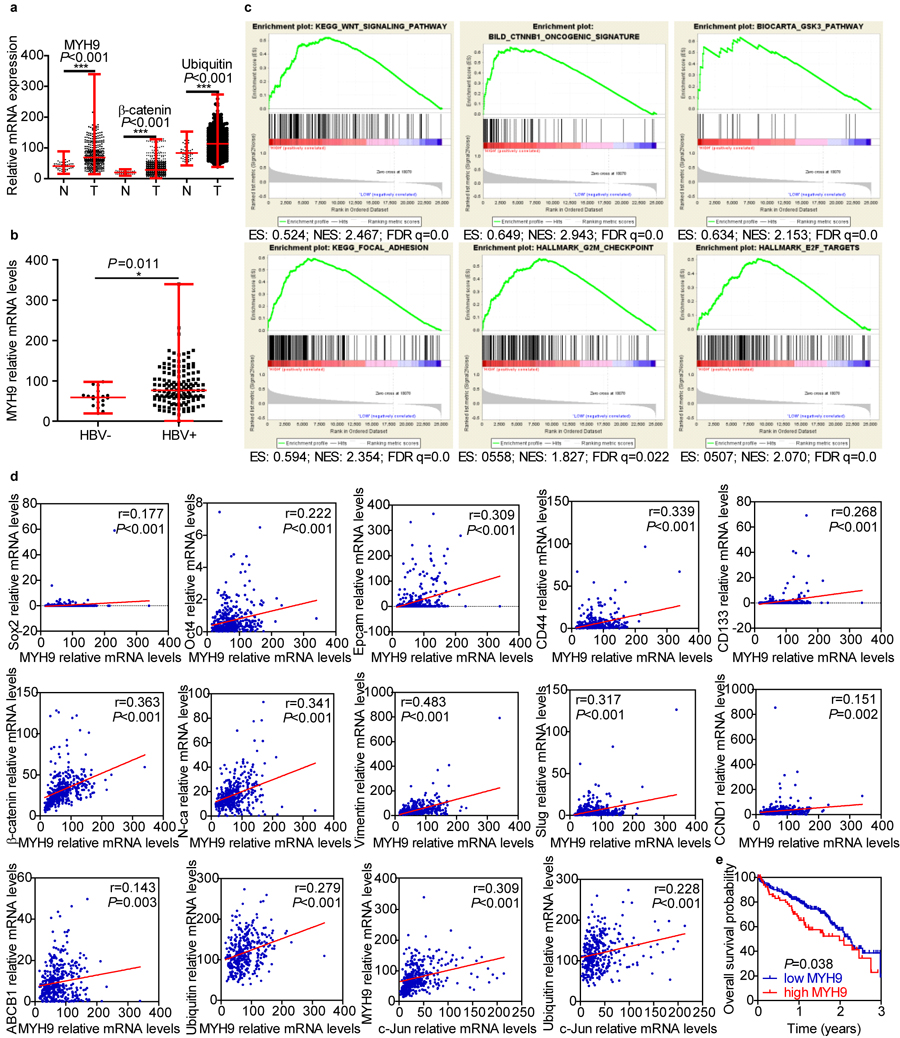


**Supplementary Fig. 6 The bioinformatics analyses of MYH9, β-catenin, and ubiquitin expression based on data from TCGA database.** (a) Comparison of MYH9, β-catenin, and ubiquitin expression between HCC and para-carcinoma tissues. (b) Comparison of MYH9 expression between HBV-negative and HBV-positive HCC tissues. (c) GSEA of MYH9 showing significant enrichment of the gene set involved in the modulation of Wnt/β-catenin/GSK3β signaling pathway, focal adhesion, and the cell cycle. (d) The relationships between CD44, CD133, Epcam, Sox2, Oct4, Slug, N-ca, vimentin, CCND1, ABCB1, ubiquitin, β-catenin, and MYH9 expression, and the relationships between MYH9, ubiquitin, and c-Jun expression. (e) Kaplan-Meier survival analyses of HCC patients according to MYH9 expression. The lines indicate median values, and the whiskers indicate minimum and maximum values (a, b).

Table S1.

| **Supplementary Table 1 Mass spectrometric analysis identified MYH9 as a protein interacted with HBX** | | | | | | |
| --- | --- | --- | --- | --- | --- | --- |
| Accession | Score | Mass | Matches | Sequences | emPAI | Protein description |
| [MYH9_HUMAN](http://fitgene-cell-pc/mascot/cgi/master_results_2.pl?file=20160902/F016128.dat;pr.eh=8,8p;pr.page=8;pr.per_page=1;pr.show=proteins) | 1067 | 227646 | 44 (26) | 38 (21) | 0.43 | Myosin-9 OS=Homo sapiens GN=MYH9 PE=1 SV=4 |

Table S2.

| **Supplementary Table 2 Correlation between HBX and MYH9 expression in hepatocellular carcinoma** | | | | | |
| --- | --- | --- | --- | --- | --- |
| **HBX expression** | **MYH9 expression** | | **Total** | ***Kappa*** | ***P* value** |
|  | **Low** | **High** |  |  |  |
| **Low** | 26 (65.0%) | 14 (35.0%) | 40 | 0.254 | 0.012 |
| **High** | 20 (38.5%) | 32 (61.5%) | 52 |
| **Total** | 46 | 46 | 92 |  |  |

Table S3.

| **Supplementary Table 3 Correlation between MYH9 and nuclear β-catenin expression in hepatocellular carcinoma** | | | | | |
| --- | --- | --- | --- | --- | --- |
| **MYH9 expression** | **Nuclear β-catenin expression** | | **Total** | ***Kappa*** | ***P* value** |
|  | **Negative** | **Positive** |  |  |  |
| **Low** | 40 (84.2%) | 6 (15.8%) | 46 | 0.309 | 0.003 |
| **High** | 28 (64.8%) | 19 (35.2%) | 47 |
| **Total** | 68 | 25 | 93 |  |  |

Table S4.

| **Supplementary Table 4 The sequences used in this study.** | | | |
| --- | --- | --- | --- |
| c-Jun | 1 | Sense | 5’ GGCACAGCUUAAACAGAAA dTdT 3’ |
|  |  | Antisense | 3’ dTdT CCGUGUCGAAUUUGUCUUU 5’ |
|  | 2 | Sense | 5’ CGCAGCAGUUGCAAACAUU dTdT 3’ |
|  |  | Antisense | 3’ dTdT GCGUCGUCAACGUUUGUAA 5’ |
| HBX | 1 | Sense | 5’ GCACUUCGCUUCACCUCUG dTdT 3’ |
|  |  | Antisense | 3’ dTdT CGUGAAGCAAGUGGAGAC 5’ |
|  | 2 | Sense | 5’ -GGUCUUACAUAAGAGGACU dTdT 3’ |
|  |  | Antisense | 3’ dTdT CCAGAAUGUAUUCUCCUGA 5’ |
| MYH9 | 1 | Sense | 5’ GCAAGCUGCCGAUAAGUAU dTdT 3’ |
|  |  | Antisense | 3’ dTdT CGUUCGACGGCUAUUCAUA 5’ |
|  | 2 | Sense | 5’ GCAAAUUCAUUCGCAUCAA dTdT 3’ |
|  |  | Antisense | 3’ dTdT CGUUUAAGUAAGCGUAGUU 5’ |
| β-catenin | 1 | Sense | 5’ GAUGGUGUCUGCUAUUGUA dTdT 3’ |
|  |  | Antisense | 3’ dTdT CUACCACAGACGAUAACAU 5’ |
|  | 2 | Sense | 5’ GGACAAGGAAGCUGCAGAA dTdT 3’ |
|  |  | Antisense | 3’ dTdT CCUGUUCCUUCGACGUCUU 5’ |
| GSK3 β | 1 | Sense | 5’ AAGAAUCGAGAGCUCCAGAUC dTdT 3’ |
|  |  | Antisense | 3’ dTdT UUCUUAGCUCUCGAGGUCUAG 5’ |
|  | 2 | Sense | 5’ AAGUAAUCCACCUCUGGCUAC dTdT 3’ |
|  |  | Antisense | 3’ dTdT UUCAUUAGGUGGAGACCGAUG 5’ |
| MYH9 shRNA |  | Sense | 5’CGCGTCCCCGCAAACCTCGAGAAGGCAATTCAAGAGATTGCCTTCTCGAGGTTTGCTTTTTGGAAAT 3’ |
|  |  | Antisense | 5’CGATTTCCGCAAACCTCGAGAAGGCAATCTCTTGAATTGCCTTCTCGAGGTTTGCGGGGA 3’ |

Table S5.

| **Supplementary Table 5 The primers used in this study.** | | |
| --- | --- | --- |
| Primers name |  | Sequence (5’-3’ ) |
| c-Jun | Forward | CCTGCGTCTTAGGCTTCTCC |
|  | Reverse | GCTCGCCCAAGTTCAACAA |
| HBX | Forward | CACTTCGCTTCACCTCTGC |
|  | Reverse | TCGGTCGTTGACATTGCTG |
| MYH9 | Forward | AGTTTGTCTCGGAGCTGTGG |
|  | Reverse | GGTTCGTGTTCCTCAGCGTA |
| GSK3 β | Forward | GTCCGATTGCGTTATTTC |
|  | Reverse | AAGAGGTTCTGCGGTTTA |
| β-catenin | Forward | GGCCCAGAATGCAGTTCGCCTT |
|  | Reverse | AATGGCACCCTGCTCACGCA |
| Ubiquitin | Forward | TCATAAGACTCGGCCTTAGAAC |
|  | Reverse | ACAAGAACTGCGACCCAAA |
| β actin | Forward | CTCGCTGTCCACCTTCCA |
|  | Reverse | ACCTTCACCGTTCCAGTTTT |
| promoter of MYH9-A | Forward | GGAAACGGGATTTCTTCA |
|  | Reverse | GAGGGTTTCACATCTGCTCT |
| promoter of MYH9-B | Forward | CCTTTCTTTTGTTGGAGC |
|  | Reverse | CTTCTGTCGAACATTACATG |
| promoter of MYH9-C | Forward | CACCCTATTTGTGCAGTT |
|  | Reverse | CCTTTTCTACATTTGGTCTC |
| promoter of UBC-A | Forward | GAGTCACCCAAGTCCCGTCCTA |
|  | Reverse | AGCGAGCGTCCTGATCCTTC |
| promoter of UBC-B | Forward | CTGCTGCAAGCCTTGTGA |
|  | Reverse | TTTCCTCCTCCTGTCCTCC |
| promoter of UBC-C | Forward | AACCTAAACCCTGCTGACC |
|  | Reverse | CCTCTGTCCCTTCTCCATT |

Table S6.

| **Supplementary Table 6 The sequences used in Electrophoretic mobility shift assay (5’-3’).** | | | |
| --- | --- | --- | --- |
| MYH9 | probes | wild type | AGGGCAGCGTCTGTGACTGACTCATTATGAGCTCACATAATCATCATGACATCATCATAGCAAATACCTAGATGGACAGTGTTGTGAATCAGGCACGGGAAC |
|  | competitors | wild type | AGGGCAGCGTCTGTGACTGACTCATTATGAGCTCACATAATCATCATGACATCATCATAGCAAATACCTAGATGGACAGTGTTGTGAATCAGGCACGGGAAC |
|  |  | site 1 mutant | AGGGCAGCGTAGAGAGTCCTCTAGGTATGAGCTCACATAATCATCATGACATCATCATAGCAAATACCTAGATGGACAGTGTTGTGAATCAGGCACGGGAAC |
|  |  | site 2 mutant | AGGGCAGCGTCTGTGACTGACTCATTATGAGCTCACATAATCATCCCACACGTCGTCCAGCAAATACCTAGATGGACAGTGTTGTGAATCAGGCACGGGAAC |
|  |  | site 3 mutant | AGGGCAGCGTCTGTGACTGACTCATTATGAGCTCACATAATCATCATGACATCATCATAGCAAATACCTAGATGGACAAGAGGACACCGAGAGCACGGGAAC |
|  |  | all sites mutant | AGGGCAGCGTAGAGAGTCCTCTAGGTATGAGCTCACATAATCATCCCACACGTCGTCCAGCAAATACCTAGATGGACAAGAGGACACCGAGAGCACGGGAAC |
| Ubiquitin | probes | wild type | GGGACTTGGGTGACTCTAGGGCACTGGTATGTCACAAAGCTGGTGACTCAGCCTTTAAACACTGAAAACGGATCCAGTGACTCATCCCGATTCTTGCACA |
|  | competitors | wild type | GGGACTTGGGTGACTCTAGGGCACTGGTATGTCACAAAGCTGGTGACTCAGCCTTTAAACACTGAAAACGGATCCAGTGACTCATCCCGATTCTTGCACA |
|  |  | site 1 mutant | GGGACTTGGGGCGGAGAAGGGCACTGGTATGTCACAAAGCTGGTGACTCAGCCTTTAAACACTGAAAACGGATCCAGTGACTCATCCCGATTCTTGCACA |
|  |  | site 2 mutant | GGGACTTGGGTGACTCTAGGGCACTGGTATGTCACAAGAACAAATGGAAGACCTTTAAACACTGAAAACGGATCCAGTGACTCATCCCGATTCTTGCACA |
|  |  | site 3 mutant | GGGACTTGGGTGACTCTAGGGCACTGGTATGTCACAAAGCTGGTGACTCAGCCTTTAAACACTGAAAACGGGGAAGAACGTTAGATTTAGTTCTTGCACA |
|  |  | all sites mutant | GGGACTTGGGGCGGAGAAGGGCACTGGTATGTCACAAGAACAAATGGAAGACCTTTAAACACTGAAAACGGGGAAGAACGTTAGATTTAGTTCTTGCACA |
